# Pre-aging of the Olfactory Bulb in Major Depression With High Comorbidity of Mental Disorders

**DOI:** 10.3389/fnagi.2018.00354

**Published:** 2018-11-08

**Authors:** Fabian Rottstaedt, Kerstin Weidner, Thomas Hummel, Ilona Croy

**Affiliations:** ^1^Department of Psychosomatic Medicine and Psychotherapy, Technische Universität Dresden Dresden, Germany; ^2^Smell & Taste Clinic, Department of Otorhinolaryngology, Technische Universität Dresden Dresden, Germany

**Keywords:** depression, olfactory bulb, MRI, biological markers, aging, premature

## Abstract

Recent studies suggest that accelerated aging of the brain is a neuroanatomical signature of the state of mental diseases. In major depression, this pre-aging effect is negatively associated with the duration since the first onset of the disease. The olfactory bulb (OB) shrinks with age in healthy subjects and patients with mental diseases show reduced OB volumes, especially those with major depression. It is unclear whether this OB reduction in mental diseases resembles a pre-aging process and whether it is associated to the duration since the onset of the mental disease. To this aim, we investigated OB volume in 73 patients (mean-age 40.4 years, SD = 12.1 years, 57 women) with major depression and mixed comorbid mental diseases (diagnoses ranged from 1 to 6, median: 3) and 51 healthy controls (mean-age 39.2 years, SD = 13.0 years, 26 women) matched for age and sex. Patient’s first onset of disease ranged from 15 to 53 years (mean 24.2 years). All participants underwent structural MR imaging with a spin-echo T2-wheighted sequence covering the anterior and middle segments of the skull base. All results were corrected for total intracranial volume (TIV) and sex. Individual OB volume was calculated by planimetric manual contouring and the pronounced diameter change in transition from bulb to tract was used as the distal demarcation of the OB. Inter-rater correlation between two independent persons analyzing the data was high (IRC = 0.81, *p* < 0.005). An age-dependent decline of the OB volume was confirmed in healthy controls (*r* = −0.34, *p* < 0.05). However, this pattern was altered in patients where the OB volume was not related to age, but to the duration since the onset of the mental disease (*r* = −0.25, *p* < 0.05). This association remained stable when controlling for age. Additionally, analyses of age sub-groups revealed that the association between duration since the onset of the mental disease and OB volume was mainly driven by the group aged 50 years and above (*r* = −0.68; *p* < 0.01). We conclude that there are time windows where the OB volume is susceptible for the effects of a mental disease, e.g., depression. These effects result in cumulative pre-aging in the OB in older patients with mental diseases.

## Introduction

Throughout human life, the brain’s neural architecture undergoes a steady transformation. Whereas the early years from birth to adolescence are determined by the interplay of neural growth and differentiation (Shaw et al., 2008), adulthood is mostly characterized by depletion (Fjell et al., 2013). Especially after midlife, brain weight (Skullerud, 1985) and whole brain volume decrease (Scahill et al., 2003; Hedman et al., 2012) and equally the volume of most brain structures shrinks with increasing age (Allen et al., 2005; Raz and Rodrigue, 2006). Accordingly, the olfactory bulb (OB) shows its peak volume around the age of 40 years from where it linearly decreases with age (Buschhüter et al., 2008). In line with this, olfactory functioning shows the same trajectory, peaking around the age of 40 years decreasing thereafter (Buschhüter et al., 2008). Most investigation of the aging brain are based on the frontal cortex and hippocampus (Fjell et al., 2014), showing that over the life span both regions are particularly vulnerable to age and undergo comparable decline in GM volume (Fjell et al., 2014).

Depressed patients show alterations that are similar to the described processes of aging. Those affect particularly brain networks involving limbic and prefrontal regions. Similar to aging, the typical GM reduction patterns in depression especially concern the hippocampus and prefrontal cortex (Bora et al., 2012; Sacher et al., 2012; Singh et al., 2013). Interestingly, depression is also related to premature reduction of OB volume (Negoias et al., 2010; Croy et al., 2013) which was suggested as biological marker for the disease (Kohli et al., 2016; Croy and Hummel, 2017; Rottstaedt et al., 2018). We hence aimed to investigate the association of OB volume decline with age in healthy controls and depressed patients. We assumed that the age-related decline of OB volume in healthy participants is shifted to younger age in patients with depression.

## Materials and Methods

### Participants

This study was embedded in a larger design (for complete Methods and Materials information please compare; Rottstaedt et al., 2018). Of the patient cohort investigated there, only patients with diagnosed Major Depression (*n* = 73, all inpatients of the Department of Psychosomatic and Psychotherapy of the Dresden University Hospital) were included in this investigation. Structured anamnestic interviews (German version of the SCID-I; Wittchen, 1997) performed by trained psychotherapists had previously been completed. The patient group included 57 females and 16 males, aged between 19 and 62 (M ± SD = 40.4 ± 12.1) years (compare Table 1 for further demographic and illness-related parameters). Diagnoses included unipolar depression or recurrent depressive disorder (*N* = 73), anxiety disorders (*N* = 47) somatoform disorders (*N* = 20), posttraumatic stress disorder (*N* = 37), substance abuse (*N* = 9) and eating disorders (*N* = 18; compare supplementary information for individual data) and hence the median of the number of overall diagnosed mental disorders was 3 (range from 1 to 6 diagnoses; compare Supplementary Table S1 for an overview of all patients and their diagnoses). The majority of patients received medical treatment (compare Supplementary Table S1). Fifty-one age and sex matched healthy controls (26 females and 25 males; 20–69 years; M ± SD = 39.16 ± 13.0 years) were recruited who were required not to meet criteria of a mental disorder (confirmed by the Patient Health Questionnaire; Spitzer et al., 1999). Exclusion criteria were concomitant nasal pathology (e.g., severe septal deviation, sinonasal disease) or potential brain pathology which was ascertained by detailed medical interview and whole brain magnetic resonance imaging (MRI) scans.

**Table 1:** Descriptive characteristics of the patient and control group.

	Patients (*N* = 73)		Healthy controls (*N* = 51)		Comparison
	Mean	SD	Mean	SD
Age (*y*)	40.4	12.1	39.2	13.0	*t*_(124)_ = −0.68
OB_left (mm^3^)	58.1	15.6	67.0	16.7	*t*_(124)_ = 3.1**
OB_right (mm^3^)	61.9	19.3	71.2	17.1	*t*_(124)_ = 3.1**
OB_best (mm^3^)	64.2	18.5	74.2	17.5	*t*_(124)_ = 3.3**
Total Intracranial Volume	1568.0	274.4	1628.6	235.7	*t*_(124)_ = 1.2
Olfactory threshold	9.4	3.6	10.2	3.3	*F*_(2,124)_ = 1.1
Olfactory identification	26.7	2.4	27.0	2.6	*F*_(2,124)_ = 0.4
BDI	31.4	12.2	2.8	2.8	*t*_(124)_ = −20.6***
Number of diagnoses	3.0	1.5			
Sex	*m* = 17 (22.6%)		*m* = 25 (49%)		$X_{\left(1,124\right)}^{2}$ = 10.4**
	*f* = 57 (77.4%)		*f* = 26 (51%)		
	*n*		%	

Age is shown in years, volumes of OB and Total Intracranial Volume are shown in mm^3^, *n*, number; %, percentage; OB, olfactory bulb; OB_left_, volume of left OB, OB_right_, volume of right OB, OB_best_, greater volume of left or right OB, BDI, score in Beck’s Depression Inventory; **p < 0.01, ***p < 0.001.

The groups did not differ in terms of age (*t*_(124)_ = 0.68, *p = 0.5*0). Sex distribution appeared not to be equal between groups ($X_{\left(1,124\right)}^{2}$ = 10.8, *p* < 0.01). Hence all statistical analyses were controlled for sex differences.

### Ethics Statement

The study followed the Declaration of Helsinki on Biomedical Research Involving Human Subjects and was approved by the local Ethics Committee (EK 51022015). All participants provided written informed consent.

### Magnetic Resonance Imaging Procedures

MRI scans were performed using an 8-channel phased-array head coil (3T Siemens Magnetom Verio scanner; Siemens Healthcare, Erlangen, Germany).

In order to obtain OB volume measures, a fast spin-echo T2-wheighted sequence covering the anterior and middle segments of the skull base was acquired (TR = 8,090 ms; TE = 97 ms; voxel size 2 × 2 × 2 mm^3^; flip angle 123°, in total 36 contiguous slices of 2 mm thickness, coronal orientation with no gap).

### Statistical Analysis

Data was analyzed using SPSS 21 for Windows (SPSS Inc., Chicago, IL, USA). AMIRA 3D visualization and modeling system (Visage Imaging, Carlsbad, CA, USA) was used to calculate OB volumes.

#### Calculation of OB Volumes

**Figure 1:**
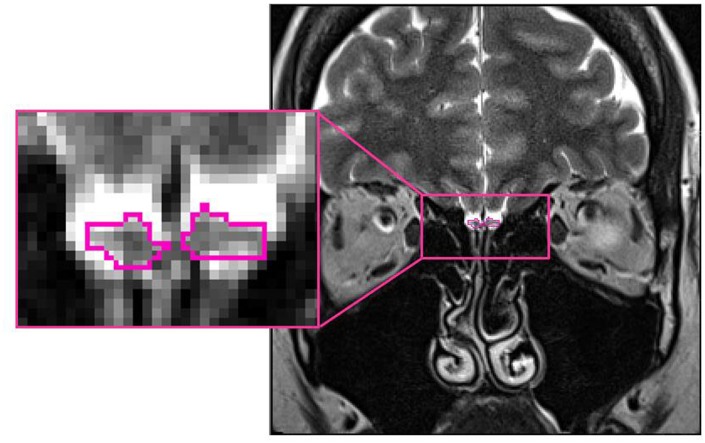
Position of the olfactory bulb (OB; encircled in pink) in the human brain (healthy control subject: 26 years old, female; MNI space: *Y* = +40).

Based on manual segmentation of the acquired T2-weighted coronal data, all OB volumes were calculated by the same experimenter (FR) blinded to the diagnosis of the participant. The pronounced diameter change in transition from bulb to tract was used as the proximal demarcation of the OB. On each coronal slice, right and left OB’s shape was outlined manually and OB volumes were calculated by planimetric manual contouring (surface in mm^2^). All surfaces were then added and multiplied by 2 (2-mm slice thickness) to obtain an estimated overall volume (compare Figure 1). The volume of the left and right OB was calculated for each participant. The larger of the two volumes was then used for all further analyses. Hence the term OB volume refers to “best OB volume.” This approach of calculating and analyzing OB volumes has previously shown to be highly reliable and accurate (Yousem et al., 1997; Hummel et al., 2015). Inter-rater correlation between two independent persons analyzing the OB volume was high (IRC = 0.81, *p* < 0.005).

#### Correlation Analysis

Pearson-correlation coefficients were computed in healthy controls and depressed patients separately to assess associations between age and OB volume. In depressed patients Pearson-correlation coefficients for the association between OB volume and the duration since the first onset of mental disease were computed and additionally controlled for age. The first onset of mental disease was taken from the documentations of the structured anamnestic interviews (German version of the SCID-I; Wittchen, 1997) where a detailed inquiry of episodes of mental disease is essential.

Furthermore, we divided the group of depressed patients into three different age groups of young (18–34 years), middle-aged (35–49 years) and old (above 50 years) individuals. For every group we computed Pearson-correlation coefficients for the association between OB volume and the duration since the first onset of the mental disease separately.

All analyses were controlled for six medical conditions (medication in general, Antidepressants, Neuroleptics, Antiepileptics, Soporifics/Tranquilizer, other drugs) by introducing them sequentially as confounding variables in a partial correlation design.

#### Between Group Analysis

OB volumes were compared between patients and controls using ANCOVA with sex and total intracranial volume (TIV) as covariate. Sex was chosen as covariate because women exhibit a smaller OB volume than men (Buschhüter et al., 2008) and sex distribution was not equal between groups; TIV was chosen as covariate as it was positively associated with OB volume (*r* = 0.24; *p* < 0.01).

All analyses were controlled for six medical conditions (medication in general, Antidepressants, Neuroleptics, Antiepileptics, Soporifics/Tranquilizer, other drugs) by introducing them sequentially as confounding variables in a partial correlation design.

## Results

**Figure 2:**
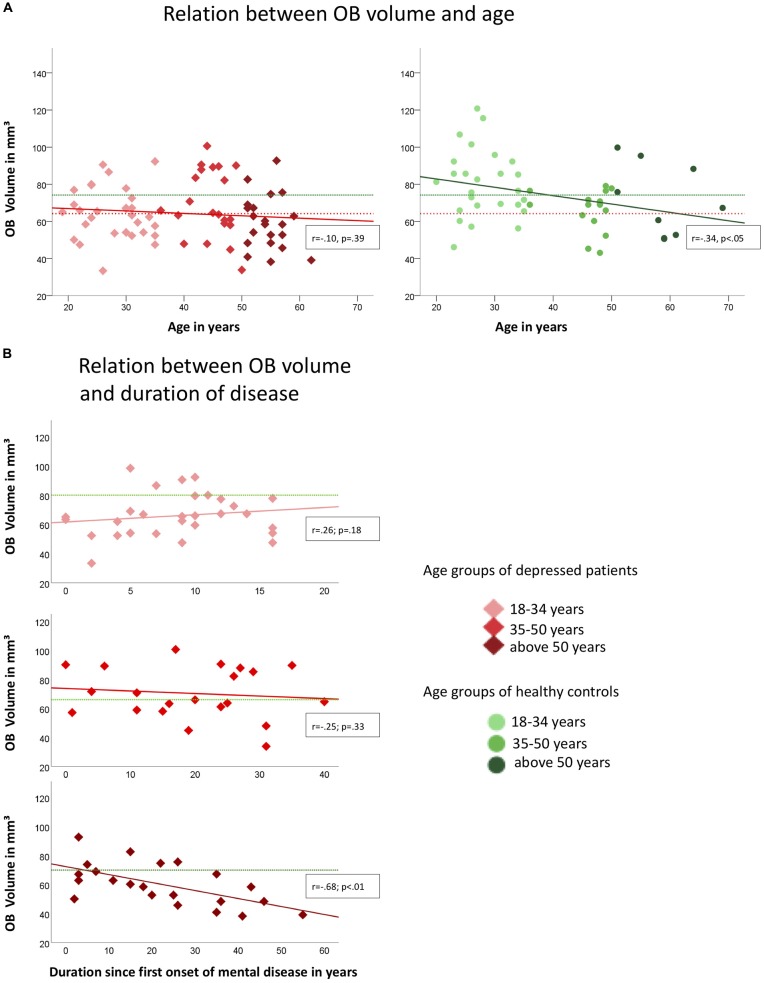
Associations of OB volume with age **(A)** and duration since first onset of mental disease **(B)** in depressed patients (indicated by red diamonds) and healthy controls (indicated by green dots) respectively. Colored graphs show Pearson-correlations of the depicted parameters, dotted lines in **(A)** indicate the mean OB volume for healthy controls (green; mean = 74.24 mm^3^) and depressed patients (red; mean = 64.27 mm^3^); in **(B)** green dotted lines indicate the mean OB volume for the corresponding age-group of healthy controls (upper diagram, 18–34 years: mean = 80.2 mm^3^, mid diagram, 35–50 years: mean = 66.2 mm^3^, lower diagram, above 50 years: mean = 71.0 mm^3^); OB Volumes are shown in mm^3^, age and duration are displayed in years.

In healthy controls, OB volume was negatively associated with age (*r* = −0.34, *p* < 0.05; compare Figure 2A) which was not the case in depressed patients (*r* = −0.10; *p* = 0.39). However, when controlling for age OB volume was related to the duration since the first onset of mental disease (*r* = −0.25, *p* < 0.05; Figure 2B). In the group of patients, the oldest individuals (aged 50 years and above) showed the strongest association between the duration since the first onset of the mental disease and OB volume (*r* = −0.68; *p* < 0.01) when controlling for age, whereas for the youngest (aged between 18 years and 34 years; *r* = 0.26; *p* = 0.18) and middle-aged (aged between 34 years and 50 years; *r* = −0.25; *p* = 0.33) patients the associations did not reach significance. In the oldest age-group, a manifestation of OB volume reduction was evident after 5 years of mental disease (compare Figure 2). Inclusion of medication did not change the results.

Depressed patients exhibited smaller OB volumes than healthy controls (*F*_(2,124)_ = 6.0; *p* < 0.05; for further comparisons of the two groups please compare Table 1). Inclusion of medication did not change the results.

## Discussion

An age-dependent decline of the OB volume had been shown in healthy people (Yousem et al., 1998; Buschhüter et al., 2008). However, this pattern was altered in patients where OB volume was not related to age, but to the duration since the first onset of the mental disease.

In detail, the OB volume showed accelerated decrease with the duration of the mental disease in patients of the oldest age-group in the sample. Notably, the data suggests that this acceleration was evident after about 5 years of duration of the mental disease. No such association was found for the middle-aged subgroup. The youngest patient sub-group showed highly reduced OB volumes compared to the respective healthy control group. However, this group still showed a trend towards an increase of the OB volume over time which can also be seen in healthy controls at this age in other studies (Yousem et al., 1998; Buschhüter et al., 2008). We conclude that there are time windows where the OB volume is susceptible for the effects of depression, namely the young and the old age. This is in line with the observation of developmental time windows of the human brain (Lupien et al., 2009) which implies that certain brain areas show increased vulnerability during specific development stages.

Two mechanisms are possible here: (a) reduced OB volume could be a pre-existing factor of the mental disease and hence indicate increased vulnerability for a mental disease (as already formulated by Croy and Hummel, 2017); and (b) The OB volume reduction could be the consequence of the mental disease. In line with the vulnerability hypothesis, the data shows that patients of the youngest age group exhibit reduced OB volumes compared to healthy controls. On the other hand, the very same result can also be interpreted as a consequence of reduced OB volume growth which happens during this time window in healthy individuals and seems diminished in patients with depression. Furthermore, the results of the group of older patients are in favor of the consequence hypothesis: in healthy aging, the OB volume starts to decrease at the age of about 40 years (Buschhüter et al., 2008). The manifestation of a mental disease increases those normal aging effects. Also known as the neurotoxicity hypotheses, this theory suggests that a long-term increase of individual stress levels leads to prolonged exposure to glucocorticoids which reduces the ability of neurons to resist insults, increasing the rate at which they are damaged by other toxic challenges or ordinary attrition (Lupien et al., 2009). In patients aged 35–49 years however, the absence of a relation between depression and OB volume could be interpreted as a higher resilience to damage of the OB during middle-age.

Gray matter volume reductions throughout the brain are well-known in depression and affect particularly brain networks involving limbic and prefrontal regions (Bora et al., 2012; Sacher et al., 2012; Singh et al., 2013). Recently, the OB volume reduction was suggested as an additional biomarker for the disease (Croy and Hummel, 2017; Rottstaedt et al., 2018). As depression is connected with increased stress levels (Liu and Alloy, 2010), risk for hypertension (Grippo and Johnson, 2009), diabetes (Talbot and Nouwen, 2000; Ali et al., 2006) and reduced physical activity (Camacho et al., 1991; Teychenne et al., 2008)—conditions known to drive brain aging processes (Raz and Rodrigue, 2006; Lupien et al., 2009; Fjell et al., 2013)—the neural alterations usually reported in depression could also be seen as manifestations of accelerated brain aging. Supporting this assumption, it could be shown that especially early-onset depression is associated with accelerated brain aging (Koutsouleris et al., 2013) and that depression duration rather than age predicts hippocampal volume loss (Sheline et al., 1999). Our findings point in the same direction and we assume that the manifestation of depression provokes cumulative pre-aging in the OB, especially in older subjects.

To sufficiently explore which of the provided theories best explains the association between OB volume reduction and depression, long-term studies are necessary. It is possible that both mechanisms—preterm aging as a consequence of depression and increased vulnerability—could work together in a vicious circle and hence are not mutually exclusive (Lupien et al., 2009; Croy and Hummel, 2017): exposure to stress during developmental periods of certain brain regions might alter their development and lead to increased vulnerability to mental disorders (de Kloet et al., 2005; Cohen et al., 2006), e.g., depression. On the other hand long-term exposure to stress during periods of mental disease could also affect brain organization (Frodl et al., 2008). For the OB that would mean cumulative volume reduction that accelerates the decline seen in older individuals.

## Limitations

There are two major limitations when interpreting the results: (1) the presented data is cross-sectional. To sufficiently explore aging trajectories, longitudinal designs are necessary; and (2) The group sizes of the investigated age-sub-groups are rather small. Hence, the interpretations of the results concerning those sub-groups should be treated with care. Investigations in larger cohorts are necessary to finally confirm or disprove the divided interpretations.

## Author Contributions

FR: data acquisition, data analysis and interpretation, drafting the article. KW and TH: substantial contribution to design of the study, critical revision of article. IC: design of the study, data interpretation, revision of article for important intellectual content.

## Conflict of Interest Statement

The authors declare that the research was conducted in the absence of any commercial or financial relationships that could be construed as a potential conflict of interest.
