# Supplementary material for: Pre-aging of the Olfactory Bulb in Major Depression With High Comorbidity of Mental Disorders
Source: Front Aging Neurosci. 2018 Nov 8;10:354. doi: 10.3389/fnagi.2018.00354 (PMC6235905; doi:10.3389/fnagi.2018.00354)
Supplement: Supplementary file 1 [file Table_1.DOC]

**Supplementary Information**

Supplementary Table 1: Sex, age and psychiatric diagnoses of the patient group

| Code | Age | Sex | Diagnoses | | | | | |
| --- | --- | --- | --- | --- | --- | --- | --- | --- |
| 1^1; 5^ | 1 | 1 | F33.1 | F45.2 | F10.1 | F42.2 |  |  |
| 2^1^ | 2 | 1 | F33.2 | F40.1 | F43.1 | F10.2 |  |  |
| 3 | 3 | 1 | F33.1 |  |  |  |  |  |
| 4^1; 5^ | 3 | 1 | F43.1 | F33.1 | F40.1 | F40.2 |  |  |
| 5^1; 5^ | 1 | 1 | F33.1 | F10.1 | F40.01 | F40.1 |  |  |
| 6^1; 5^ | 2 | 1 | F33.2 | F40.01 |  |  |  |  |
| 7^1^ | 2 | 1 | F33.2 | F41.0 | F40.1 | F10.20 |  |  |
| 8^1^ | 3 | 1 | F32.0 | F45.1 |  |  |  |  |
| 9^5^ | 2 | 1 | F33.2 |  |  |  |  |  |
| 10^1; 5^ | 3 | 1 | F33.1 |  |  |  |  |  |
| 11^1^ | 1 | 1 | F33.1 | F45.4 |  |  |  |  |
| 12^1; 5^ | 1 | 1 | F33.0 | F50.0 | F60.6 |  |  |  |
| 13 | 2 | 1 | F43.1 | F32.2 | F45.4 |  |  |  |
| 14^1; 2; 5^ | 1 | 1 | F45.2 | F33.1 |  |  |  |  |
| 15^1; 2; 5^ | 2 | 1 | F33.1 | F40.1 | F40.01 |  |  |  |
| 16^1; 5^ | 1 | 1 | F33.0 | F40.1 | F43.1 | F10.2 |  |  |
| 17^1; 2; 5^ | 3 | 2 | F43.1 | F33.2 | F42.1 | F40.2 | F50.9 |  |
| 18^1; 2; 5^ | 3 | 2 | F43.1 | F33.1 |  |  |  |  |
| 19^1; 2^ | 1 | 2 | F43.1 | F33.2 | F40.2 | F50.0 | F50.2 |  |
| 20^1; 5^ | 2 | 2 | F43.1 | F33.2 | F45.0 |  |  |  |
| 21^1; 2; 5^ | 2 | 2 | F32.1 | F41.0 | F40.1 | F50.2 |  |  |
| 22^1; 2^ | 1 | 2 | F50.0 | F33.1 |  |  |  |  |
| 23^5^ | 2 | 2 | F43.1 | F40.01 | F33.1 | F40.2 |  |  |
| 24^2; 4; 5^ | 2 | 2 | F43.1 | F33.1 | F41.0 | F40.2 |  |  |
| 25^1; 2; 5^ | 3 | 2 | F40.1 | F43.1 | F33.1 | F40.01 |  |  |
| 27^1; 2; 5^ | 1 | 2 | F33.2 | F40.01 |  |  |  |  |
| 27 | 1 | 2 | F43.1 | F33.1 | F40.0 | F40.1 | F40.2 |  |
| 28^5^ | 1 | 2 | F43.1 | F33.1 | F50.2 | F40.1 |  |  |
| 29^1; 5^ | 1 | 2 | F43.1 | F33.2 |  |  |  |  |
| 30 | 3 | 2 | F43.1 | F33.1 | F40.01 | F45.1 | F40.1 |  |
| 31^1; 2; 5^ | 3 | 2 | F40.01 | F33.2 | F43.1 |  |  |  |
| 32^1; 5^ | 3 | 2 | F43.1 | F40.1 | F33.2 | F45.4 | F40.2 |  |
| 33^1; 3; 5^ | 2 | 2 | F43.1 | F40.1 | F33.2 | F42.1 | F40.2 |  |
| 34^1; 3; 5^ | 3 | 2 | F33.0 |  |  |  |  |  |
| 35^1^ | 1 | 2 | F50.0 | F43.1 | F40.00 | F33.2 |  |  |
| 36^5^ | 3 | 2 | F33.1 |  |  |  |  |  |
| 37^1; 2; 3; 5^ | 2 | 2 | F43.1 | F32.3 | F42.1 | F10.2 | F50.2 | F40.01 |
| 38^1^ | 1 | 2 | F33.1 | F40.1 | F43.1 |  |  |  |
| 39^1; 2; 5^ | 2 | 2 | F43.1 | F33.2 |  |  |  |  |
| 40 | 1 | 2 | F43.1 | F33.1 | F40.01 | F40.1 |  |  |
| 41^1^ | 1 | 2 | F45.1 | F33.0 |  |  |  |  |
| 42^1; 5^ | 2 | 2 | F43.1 | F33.2 | F40.01 | F45.4 | F40.1 |  |
| 43^1; 5^ | 2 | 2 | F43.1 | F33.2 | F40.1 |  |  |  |
| 44^1; 5^ | 3 | 2 | F50.2 | F33.2 | F45.4 | F40.1 |  |  |
| 45 | 3 | 2 | F33.0 | F45.1 | F40.00 |  |  |  |
| 46^1; 5^ | 2 | 2 | F43.1 | F33.0 |  |  |  |  |
| 47^1^ | 3 | 2 | F33.0 | F50.0 |  |  |  |  |
| 48^1; 3; 5^ | 1 | 2 | F43.1 | F33.1 | F45.4 | F41.0 |  |  |
| 49^1; 5^ | 1 | 2 | F33.1 | F45.1 | F40.01 | F40.2 | F50.00 |  |
| 50^1; 5^ | 1 | 2 | F50.0 | F32.0 |  |  |  |  |
| 51^5^ | 2 | 2 | F50.3 | F10.2 | F32.1 |  |  |  |
| 52^5^ | 1 | 2 | F41.1 | F32.1 | F50.2 |  |  |  |
| 53^5^ | 1 | 2 | F33.1 |  |  |  |  |  |
| 54 | 1 | 2 | F40.1 | F33.1 | F45.1 |  |  |  |
| 55^1; 5^ | 2 | 2 | F33.2 | F40.01 | F43.1 | F45.4 |  |  |
| 56^1; 5^ | 2 | 2 | F33.1 | F43.1 | F45.4 |  |  |  |
| 57^5^ | 3 | 2 | F43.1 | F33.1 | F45.4 | F42.1 | F40.1 |  |
| 58^1^ | 3 | 2 | F43.1 | F45.4 | F32.1 |  |  |  |
| 59^1; 2; 5^ | 2 | 2 | F33.1 | F40.1 |  |  |  |  |
| 60^1; 5^ | 3 | 2 | F41.0 | F33.1 | F43.1 |  |  |  |
| 61^1; 5^ | 3 | 2 | F43.1 | F33.2 | F42.1 |  |  |  |
| 62 | 1 | 2 | F50.0 | F32.0 | F40.2 |  |  |  |
| 63^1; 2; 5^ | 1 | 2 | F33.1 | F40.1 | F43.1 | F50.2 |  |  |
| 64^5^ | 1 | 2 | F50.2 | F32.1 |  |  |  |  |
| 65^1; 5^ | 1 | 2 | F43.1 | F42.1 | F33.2 | F40.2 | F15.2 | F16.1 |
| 66^1; 2, 4, 5^ | 3 | 2 | F33.2 | F40.00 |  |  |  |  |
| 67^1; 2; 3; 5^ | 2 | 2 | F33.1 | F42.0 | F43.1 | F20.0 | F40.1 |  |
| 68^1^ | 1 | 2 | F33.0 | F40.01 | F42.0 | F60.5 |  |  |
| 69^1; 5^ | 3 | 2 | F32.1 |  |  |  |  |  |
| 70^5^ | 1 | 2 | F40.1 | F43.1 | F32.1 |  |  |  |
| 71^5^ | 1 | 1 | F33.1 | F40.01 |  |  |  |  |
| 72^1; 2; 5^ | 3 | 2 | F32.1 | F45.1 | F42.1 |  |  |  |
| 73^1^ | 2 | 2 | F32.2 |  |  |  |  |  |

1=male, 2=female, for reasons of anonymity age is displayed by age groups: 1=18-35, 2=36-50, 3=51-65; Medication: ^1^ Antidepressant, ^2^ Neuroleptics, ^3^ Antiepileptics, ^4^ Soporifics/Tranquilizer, ^5^ Other drugs; F15.2 Mental and behavioural disorders due to use of other stimulants, including caffeine, Dependence syndrome, F10.1 Mental and behavioural disorders due to use of alcohol, harmful use, F10.2 Mental and behavioural disorders due to use of alcohol, Dependence syndrome, F16.1 Mental and behavioural disorders due to use of hallucinogens, harmful use, F20.0 Paranoid schizophrenia, F32.0 Mild depressive episode, F32.1 Moderate depressive episode, F32.2 Severe depressive episode without psychotic symptoms, F33.0 Recurrent depressive disorder, current episode mild, F33.1 Recurrent depressive disorder, current episode moderate, F33.2 Recurrent depressive disorder, current episode severe without psychotic symptoms, F40.0 Agoraphobia, F40.1 Social phobias, F40.2 Specific (isolated) phobias, F41.0 Panic disorder [episodic paroxysmal anxiety], F41.1 Generalized anxiety disorder, F42.0 Predominantly obsessional thoughts or ruminations, F42.1 Predominantly compulsive acts [obsessional rituals], F42.2 Mixed obsessional thoughts and act, F43.1 Post-traumatic stress disorder, F45.0 Somatization disorder, F45.1 Undifferentiated somatoform disorder, F45.2 Hypochondriacal disorder, F45.4 Persistent somatoform pain disorder, F50.0 Anorexia nervosa, F50.2 Bulimia nervosa, F50.3 Atypical bulimia nervosa, F60.5 Anankastic personality disorder, F60.6 Anxious [avoidant] personality disorder
